# Coupling Single Molecules to DNA-Based Optical Antennas with Position and Orientation Control

**DOI:** 10.1021/acsphotonics.4c01506

**Published:** 2024-11-19

**Authors:** Aleksandra K. Adamczyk, Fangjia Zhu, Daniel Schäfer, Yuya Kanehira, Sergio Kogikoski, Ilko Bald, Sebastian Schlücker, Karol Kołątaj, Fernando D. Stefani, Guillermo P. Acuna

**Affiliations:** † Department of Physics, 27211 University of Fribourg, Fribourg CH-1700, Switzerland; ‡ Department of Chemistry and Center of Nanointegration Duisburg-Essen (CENIDE) & Center of Medical Biotechnology (ZMB), 27170 University of Duisburg-Essen, 45141 Essen, Germany; § Institute of Chemistry, 26583 University of Potsdam, 14476 Potsdam, Germany; ∥ Centro de Investigaciones en Bionanociencias (CIBION), Consejo Nacional de Investigaciones Científicas y Técnicas (CONICET), C1425FQD Ciudad Autónoma de Buenos Aires, Argentina; ⊥ Departamento de Física, Facultad de Ciencias Exactas y Naturales, Universidad de Buenos Aires, C1428EHA Ciudad Autónoma de Buenos Aires, Argentina; # Swiss National Center for Competence in Research (NCCR) Bio-inspired Materials, University of Fribourg, CH-1700 Fribourg, Switzerland

**Keywords:** nanophotonics, plasmonics, single-molecule fluorescence, single-photon sources, DNA nanotechnology, optical antennas

## Abstract

Optical antennas have been extensively employed to manipulate the photophysical properties of single-photon emitters. Coupling between an emitter and a given resonant mode of an optical antenna depends mainly on three parameters: spectral overlap, relative distance, and relative orientation between the emitter’s transition dipole moment and the antenna. While the first two have already been extensively demonstrated, achieving full coupling control remains unexplored due to the challenges in manipulating at the same time both the position and orientation of single molecules. Here, we use the DNA origami technique to assemble a dimer optical antenna and position a single fluorescent molecule at the antenna gap with controlled orientation, predominately parallel or perpendicular to the antenna’s main axis. We study the coupling for both conditions through fluorescence measurements correlated with scanning electron microscopy images, revealing a 5-fold higher average fluorescence intensity when the emitter is aligned with the antenna’s main axis and a maximum fluorescence enhancement of ∼1400-fold. A comparison to realistic numerical simulations suggests that the observed distribution of fluorescence enhancement arises from small variations in the emitter orientation and gap size. This work establishes DNA origami as a versatile platform to fully control the coupling between emitters and optical antennas, trailblazing the way for self-assembled nanophotonic devices with optimized and more homogeneous performance.

## Graphical abstract

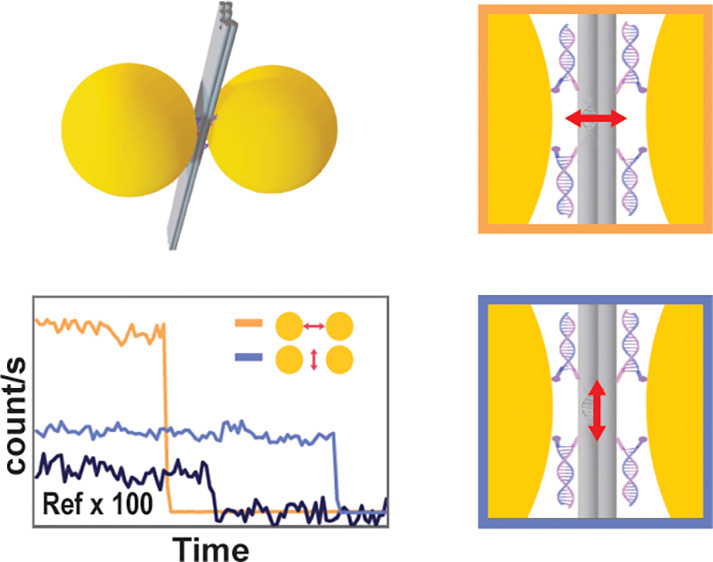

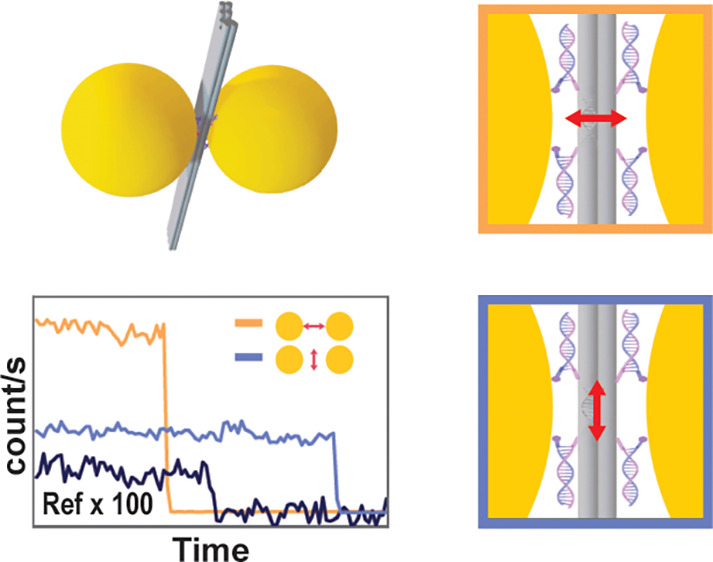

## Introduction

Optical antennas^1^ (OAs) based on metallic or dielectric nanoparticles (NPs) are one of the basic components of nanophotonic devices, acting as transducers between propagating light and localized fields, enabling a remarkable enhancement of light–matter interactions at the nanoscale.^2^ In particular, OAs have been widely demonstrated to control and manipulate the photophysical properties of single-photon emitters such as organic fluorophores and quantum dots.^3^ The interaction between a photon emitter and a given resonant mode of an OA is mainly governed by their spectral overlap, relative position, and relative orientation.^4,5^ The spectral overlap can be tuned by the choice of OA design and emitter.^6,7^ First studies of the position-dependent coupling of an emitter and an OA were done with atomic force microscopy (AFM) techniques.^8–11^ While these pioneering measurements provided valuable insight, those AFM-based techniques are challenging to implement for more complex OA geometries involving two or more NPs.^12^ Furthermore, they are hardly suited for the fabrication of antenna-emitter systems with a controlled relative position, and they provide no control over the emitter orientation. Alternatively, some works have circumvented the challenges involved in controlling the fluorophore’s orientation by first selecting molecules with a determined orientation and then, in a second step, coupling the preselected molecules to an OA.^11,13^

The advent of the DNA origami technique^14^ enabled the bottom-up self-assembly of colloidal metallic^15^ and recently dielectric^16,17^ NPs together with organic dyes and quantum dots with nanometric positional precision and stoichiometric control.^18–20^ In this way, the DNA origami technique was exploited to fabricate diverse OAs with single emitters placed at specific locations to enhance the fluorescence intensity^21,22^ and photostability,^23^ and to direct or tune the emission.^24–26^ These DNA origami-based OAs can very precisely manipulate the photophysical properties of single-photon emitters located at the hotspot as demonstrated, for example, by maximum values of fluorescence enhancement (FE) reaching 3 orders of magnitude^27^ and forward to backward directivities over 10 dB.^25^ Despite these impressive values, the overall performance of these OAs remains rather inhomogeneous, with a significant dispersion. This limitation adversely affects the efficiency and reproducibility of OAs and their implementation beyond fundamental research for quantitative applications.^28^

We hypothesize that an important factor behind the OA’s inhomogeneous overall performance in the cases reported before lies in the fact that the emitter’s transition dipole orientation was not controlled. Therefore, the OA-emitter coupling can vary from virtually suppressed to its maximum value depending on the relative orientation. However, incorporating molecules in a nanodevice with controlled position, stoichiometry, and orientation has remained an open challenge. Recently, we have demonstrated the first steps toward controlling the orientation of single fluorophores in DNA origami structures.^29^ Briefly, by engineering both the fluorophore link to DNA and the local environment, the transition dipole moment of Cy3 and Cy5 molecules can be oriented predominantly parallel or perpendicular to the host double-stranded (ds) DNA helix. Analogous results were independently obtained, reinforcing the robustness of this method.^30^ Here, we study the use of this approach to achieve full control of the coupling between a fluorophore and an OA.

## Self-Assembly of Optical Antennas with a Single Fluorophore Oriented at the Hotspot

A sketch of the DNA origami employed to self-assemble the OAs and control both the position and the orientation of a single emitter is included in Figure 1a. It consists of a two-layer 12-helix rectangular structure with dimensions of approximately 180 nm × 20 nm × 5 nm (length × width × height) with a “mast” at the center (not shown). The DNA origami structure is modified with single-stranded (ss-) DNA handles consisting of an A15 sequence to incorporate, through DNA hybridization, two 60 nm gold NPs previously functionalized with the complementary sequence T18 (further details on the DNA origami design are given in the Supplementary Note 1: Materials and Methods and Figure S1). In order to achieve more efficient and localized binding sites for the Au NPs, we extended the ssDNA staple strands of the origami of the binding region both toward their 3′ and 5′ ends with poly-A sequences. As a consequence, the Au NPs can bind to the origami in the zipper and shear configuration^31^ (For simplicity, Figure 1a shows only the zipper configuration). Under these conditions, we estimated a resulting average interparticle gap *g* = (10 ± 4) nm (further details on the dimer gap size are given in Supplementary Note 5 and Figures S1, S7, and S8). Between the Au NPs, the DNA origami hosts a single Cy5 molecule oriented either predominantly aligned with the main dimer axis (sample ∥) or perpendicular to it (sample ⊥); see zoom in Figure 1a. This is achieved by leaving no (∥) or 8 (⊥) adjacent bases unpaired from the DNA origami scaffold so that the fluorophore’s orientation is adjusted without changing its position. The OAs were then purified by gel electrophoresis and imaged by transmission electron microscopy (TEM), confirming the correct self-assembly of the structures (Figure 1b). It is worth noting that commercial Au NPs tend to differ in shape from their nominal spherical description. In addition, a control sample was fabricated using “bare” DNA origami structures with a single Cy5 without NPs and with six biotinylated modifications on the underside of the origami for sample immobilization onto glass coverslips functionalized with neutravidin.^32^

**Figure 1:**
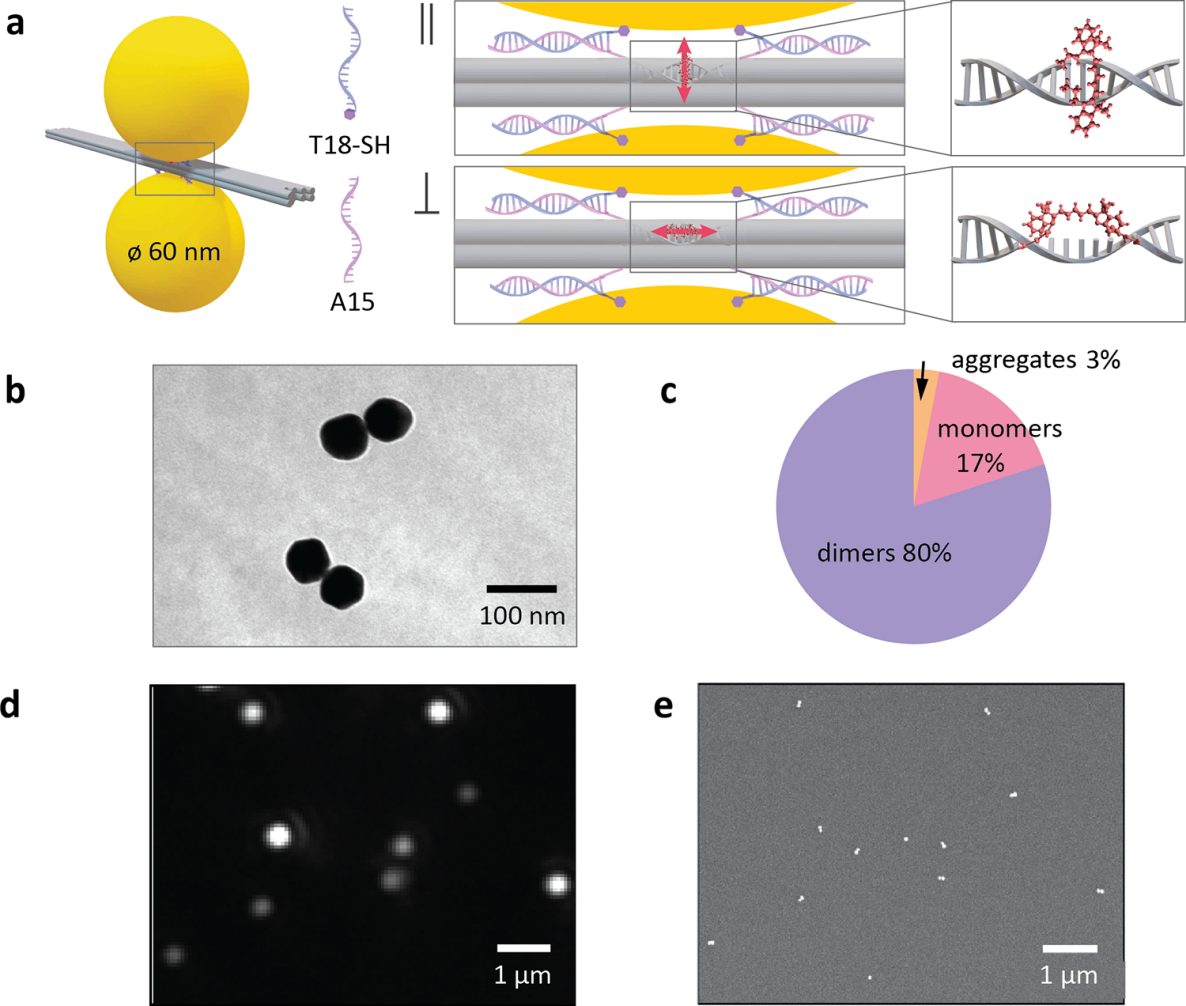
Dimer OAs with an oriented single dye at the hotspot. (a) Sketch of the DNA origami host structure and the Au NPs employed. The Au NPs are linked to the DNA origami using both a zipper and shear hybridization configuration. For the sake of clarity, only a zipper configuration is presented. The zoom highlights the gap where a doubly linked fluorophore (symbolized by a red double arrow) was positioned either perpendicular or parallel to the two 60 nm Au NPs forming the OA dimer. (b) TEM image of two OA dimers. (c) Pie chart illustrating the yield of OAs on the sample surface. (d) An exemplary image obtained with wide-field fluorescence measurements. Spots of different intensities can be already detected. (e) Corresponding SEM image of the identical area utilized for colocalization with (d) to confirm that the signal originates from dimer OAs.

The OAs and control structures were immobilized on glass coverslips at a surface density suited for single-molecule fluorescence measurements (see the Supporting Information for further details). The emission of individual OAs and control structures were measured in a wide-field microscope under circularly polarized excitation (Figure 1d). Subsequently, the samples containing the OAs were dried and transferred to a scanning electron microscope (SEM), see Figure 1e. As shown in the examples of Figure 1d,e, there is a high correlation between the detected fluorescence spots and dimer OAs imaged by SEM. The sample also contains a minor fraction of NP monomers and a small number of dimer OAs that lack a fluorescence signal. These combined SEM and fluorescence measurements allowed us to quantify the yield of functional OAs (Figure 1c) and analyze solely the fluorescence arising from dimer structures. The yield of the dimer structures was 80%, confirming the high efficiency of the DNA origami OA self-assembly process.

## Fluorescence Enhancement with the Fluorophore Oriented Parallel or Perpendicular to the Main Antenna Axis

Figure 2a shows a comparison of exemplary fluorescence intensity transients originating from single fluorophores oriented parallel and perpendicularly to the main axis of the OA dimer and from a single fluorophore in the control sample. Only traces showing single-step photobleaching were considered to ensure that the measurement corresponded to a single molecule. Molecules aligned with the main axis of the OA tend to show a significantly higher intensity. This is a direct consequence of two main effects. First, there is a more efficient excitation because the absorption dipole of the fluorophore is aligned with the gap field of the main resonant antenna mode, as schematically shown in Figure 2b. Second, the fluorophore’s quantum yield will be significantly reduced when aligned perpendicular to the main resonant antenna mode.^22^ This can be rationalized, for example, by considering the electric dipoles induced by the fluorophore on the NPs that will interact destructively with the fluorophore’s emission dipole, leading to a reduced emission into the far field.^33,34^

**Figure 2:**
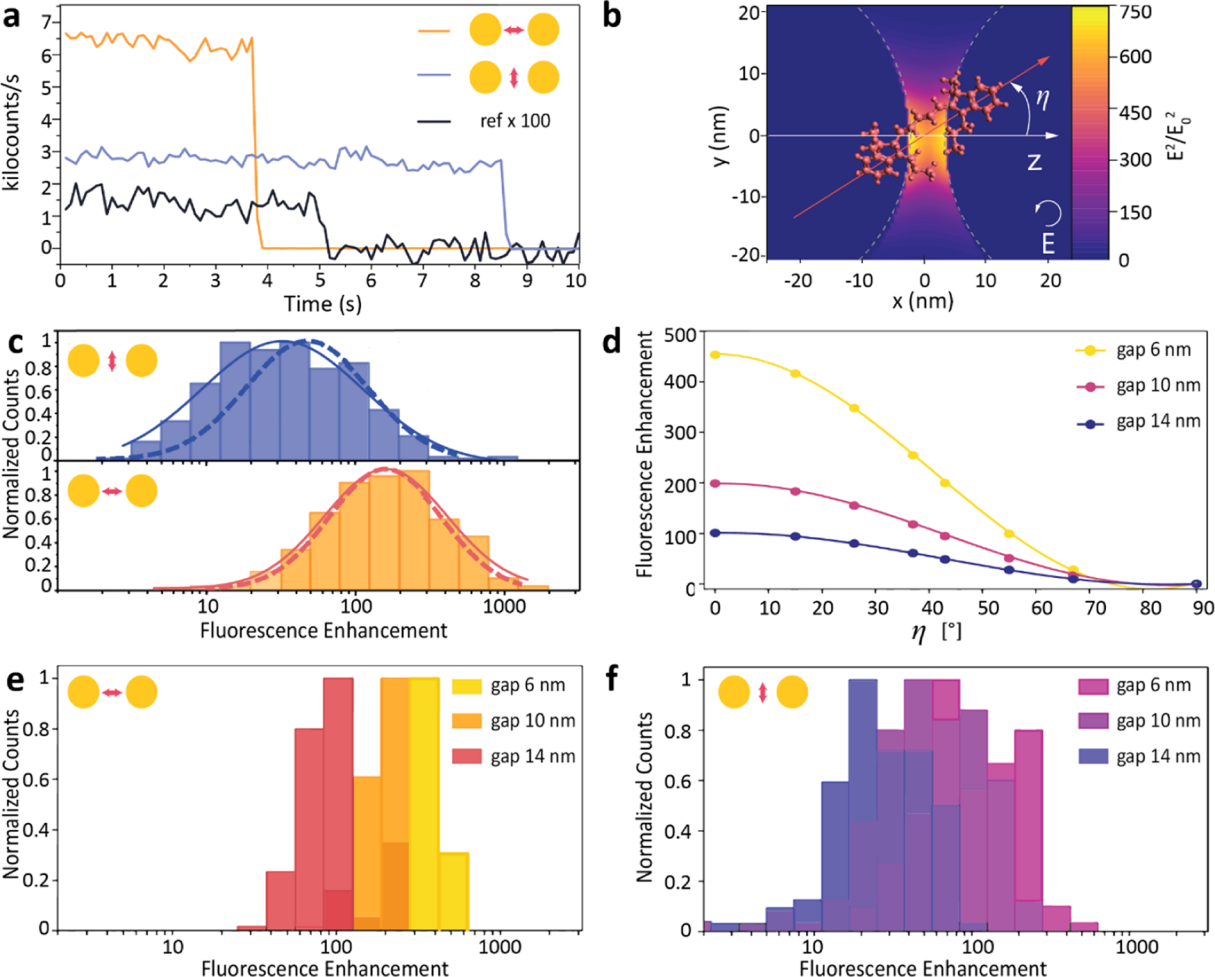
Fluorescence enhancement of OAs with oriented fluorophores at the gap: measurements and simulations. (a) Examples of fluorescence intensity traces for an OA with the fluorophore oriented parallel and perpendicular to the main axis. For comparison, a reference transient from a DNA origami containing a single Cy5 dye but no Au NPs is included. (b) Simulations of the electric field intensity enhancement for a 6 nm gap at an excitation wavelength of 640 nm with circularly polarized light. The orientation of the electric field at the gap is predominantly in line with the dimer axis (not shown). The coordinate system employed to describe the orientation of single fluorophores in the antenna is included. (c) Fluorescence enhancement histogram plot for 60 nm Au OAs dimers with a single Cy5 dye located at the hotspot oriented predominantly parallel (orange) or perpendicular (blue) to the main dimer axis. The solid lines represent a log-normal distribution fit, whereas the dashed lines correspond to numerical simulations. (d) Simulated fluorescence enhancement dependent on the fluorophore orientation for three different systems with interparticle distances equal to 6, 10, and 14 nm, respectively. The simulated fluorescence enhancement distributions for samples ∥ (e) and ⊥ (f), considering gaps of 6, 10, and 14 nm and the previously measured angular distributions.^29^

We measured fluorescence intensity transients of 404, 479, and 140 structures corresponding to samples ∥, ⊥, and the control, respectively. Figure 2c summarizes the main results, showing the distribution of fluorescence enhancement for samples ∥ and ⊥, computed as the average intensity of each trace normalized to the average fluorescence intensity of all molecules in the control sample (Figure S2). While both samples exhibit a rather broad dispersion of enhancement factors, the values obtained in the ∥ sample are significantly higher. The distributions of enhancement factors are well fit with a log-normal distribution^35^ (solid lines in Figure 2c; for more details, see Supplementary Note 2 and Table S1), retrieving the following mean fluorescence enhancement (μ) and standard error (SE): μ_∥_ = 245.3, SE_∥_ = 13.4, μ_⊥_ = 46.1 and SE_⊥_ = 3.0. Based on these results, we can conclude that by controlling the orientation of the fluorophore without affecting its position, the fluorescence signal can be increased 5-fold in average. Notably, it is worth highlighting that a maximum enhancement factor of ∼1400 was achieved for sample ∥. This is a remarkably high value for OA dimers based on 60 nm Au NPs with an average gap of 10 nm. This value translates into a fluorescence enhancement figure of merit (defined as FE × ϕ_0_) of ∼400 (considering the intrinsic quantum yield of Cy5 ϕ_0_ ∼ 0.3), which is the highest one reported to date^36^ in the visible range.

## Discussion

The results in Figure 2c clearly show that the molecular orientation can be controlled, leading to a strong effect on the coupling to the OA. However, the distributions of observed enhancement factors are relatively broad, as a result of several factors. The excitation rate might be inhomogeneous if for example, the OAs have a distribution of out-of-plane orientations. However, this is unlikely. Due to the dimensions of the NPs and the DNA origami employed together with the binding scheme, OAs will predominantly lie flat on the glass coverslip surface. The dispersion of size and shape of the NPs might also affect the homogeneity of results (see, for example, Figure 1b). To test this point, we have repeated the experiments using “superspherical” 50 nm Au NPs,^37^ see Supplementary Note 3 and Figure S3. These NPs were synthesized following the protocol included in a previous work.^38^ Based on TEM measurements, the superspherical NPs exhibited a size distribution with a diameter of 51.2 nm and a standard deviation of 2.1 nm. Our results show that OAs based on superspherical Au NPs display an overall lower enhancement as expected from the smaller size (Figure S4 and Table S2). Nonetheless, the observed distributions of fluorescence enhancement were equivalently broad to the ones obtained with commercial NPs. We therefore conclude that the dispersion of NPs size and shape does not play a crucial role in the inhomogeneity of coupling observed, which is attributed to variations in the gap size and molecular orientations.

Other factors that can lead to a dispersion of fluorescence enhancement are variations in molecular orientation and gap size. To analyze this, we performed numerical calculations to determine the expected fluorescence enhancement for a range of orientations and gaps. Figures 2d and S5 summarize the results, which show that a higher fluorescence enhancement is expected for smaller gaps and orientations close to the main axis of the OA. For the perpendicular orientation, no enhancement is expected regardless of the gap size. The observed fluorescence enhancement for the ⊥ sample, with maximum values even reaching several 100 fold, indicates that even for this configuration the molecular dipole orientation has a significant component along the main axis of the OA. This observation, together with the broad distribution of enhancement factors exposes the limitations of the approach to fix the molecular orientation, which has been characterized in a previous work.^29^

As to the gap size, standard deviations from 1 to 2.7 nm have been already observed for gaps in different DNA origami-based OAs.^21,39–41^ It is worth noting that those reported values were estimated from TEM measurements on the dried samples. However, our measurements were performed in liquid, and therefore, a different dispersion in gap sizes could be expected. In general, for dried samples, the DNA connections between NPs and DNA origami collapse and the measured gap does not solely depend on the way that the hybridization is realized but also on the interaction between the nanostructures and the specific surface, as well as on the other drying effects.^42,43^ On the other hand, the intermolecular gap in a liquid represents the actual local minimum of the thermodynamic energy of the system, which strictly depends on the hybridization scheme, temperature, and ionic strength in solution. However, measuring the actual interparticle distance in solution is challenging;^44^ therefore, we assumed the range of dimer gap size between 6 and 14 nm based on the possible binding schemes, as explained in Supplementary Note 5 and Figures S1, S7, and S8. To study the influence of gap size and molecular orientation variations on the fluorescence enhancement, we carried out numerical simulations to calculate the expected distributions of fluorescence enhancement for three gap sizes 6, 10, and 14 nm, considering the molecular angular distributions previously determined experimentally for each configuration^29^ (Figure S6; further simulation details are included in the Supplementary Note 3). Figure 2e,f shows the simulated fluorescence enhancement distributions for samples ∥ and ⊥, considering gaps of 6, 10, and 14 nm. In Figure 2c, the simulated distributions for the three gaps are summed and presented as bold dashed lines to compare with the experimental results. The numerical simulations performed with the input of the measured three-dimensional angular distributions of the fluorophores agree well with the experiments.

## Conclusions

In conclusion, this work demonstrates how DNA nanotechnology can be used to control the coupling of a single molecule to the resonant mode of an OA by simultaneously controlling the position and orientation of the molecule relative to the OAs. The strategy applied involves a double link of the fluorophore to the DNA double helix with different numbers of unpaired bases to modulate the local environment. In this way, a remarkable 5-fold increase in the average fluorescence intensity is achieved, along with an unprecedented maximum enhancement of 1400-fold. Based on control experiments using superspherical NPs and by comparing the distributions of enhancement factors to realistic simulations, we can conclude that dispersion of results arises from variations in molecular orientations and gap sizes. Looking ahead, it is worth exploring more rigid OA designs aiming to deliver more uniform gap sizes as well as new strategies for more precise control of molecular orientation. Finally, our results firmly establish the DNA origami technique as a versatile and comprehensive nanofabrication platform to control the position and orientation of different species, such as organic dyes and NPs to fully manipulate their interaction. This assembly precision holds promise for achieving OAs with maximum efficiency and reproducibility, which is a crucial step for advancing their implementation in quantitative nanophotonic applications.

## Supplementary Material



## Notes

Materials and methods employed for the assembly and measurement of the optical antennas, parameters of the distribution used to fit the data and numerical simulations of the optical antenna performance, sketch of the DNA origami employed, and results obtained with the superspherical nanoparticles (PDF)
