# Supplementary material for: Coupling Single Molecules to DNA-Based Optical Antennas with Position and Orientation Control
Source: ACS Photonics. 2024 Nov 19;11(12):5267–72. doi: 10.1021/acsphotonics.4c01506 (PMC13262693; doi:10.1021/acsphotonics.4c01506)
Supplement: Supplementary file 1 [file ph4c01506_si_001.pdf]

Supplementary Information for

Coupling single-molecules to DNA-based optical antennas with position and orientation control

Aleksandra K. Adamczyk[1], Fangjia Zhu[1], Daniel Schäfer[2], Yuya Kanehira[3], Sergio Kogikoski Jr[3], Ilko Bald[3], Sebastian Schlücker[2], Karol Kołątaj[1,6], Fernando D. Stefani[4,5*] and Guillermo P. Acuna[1,6*]*

[1] Department of Physics, University of Fribourg, Fribourg CH-1700, Switzerland.

[2] Department of Chemistry and Center of Nanointegration Duisburg-Essen (CENIDE) & Center of Medical Biotechnology (ZMB), University of Duisburg-Essen, 45141 Essen, Germany.

[3] Institute of Chemistry, University of Potsdam, 14476 Potsdam, Germany.

[4] Centro de Investigaciones en Bionanociencias (CIBION), Consejo Nacional de Investigaciones Científicas y Técnicas (CONICET), C1425FQD Ciudad Autónoma de Buenos Aires, Argentina.

[5] Departamento de Física, Facultad de Ciencias Exactas y Naturales, Universidad de Buenos Aires, C1428EHA Ciudad Autónoma de Buenos Aires, Argentina.

[6] Swiss National Center for Competence in Research (NCCR) Bio-inspired Materials, University of Fribourg, CH-1700 Fribourg, Switzerland.

Corresponding Authors

*E-mail: karol.kolataj@unifr.ch, fernando.stefani@df.uba.ar, guillermo.acuna@unifr.ch

Table of Contents

Supplementary Note 1: Materials and Methods....3
Supplementary Figure 1....6
Supplementary Figure 2....7
Supplementary Note 2: Log-normal Distributions....7
Table S1. Parameters from fits ($xc, w, \mu, \sigma,$ and SE) and measurements (N)....8
Supplementary Note 3: Super-spherical NPs results....8
Supplementary Figure 3....9
Supplementary Figure 4....10
Table S2. Parameters from fits for super-spherical NPs....10
Supplementary Note 4: Finite element method simulations....10
Supplementary Figure 5....13
Supplementary Figure 6....14
Supplementary Note 5: Consideration of the interparticle gap....14
Supplementary Figure 7....14
Supplementary Figure 8....16
References....18

Supplementary Note 1: Materials and Methods

DNA origami design and folding

The square-lattice DNA origami structure was designed using CaDNAno[1], and it is accessible at nanobase.org[2]. The 7249 bases scaffold (M13mp18, Bayou Biolabs LLC) and 242 synthetic staple strands were folded in 1× TAE (Alfa Aesar, #J63931) and 12 mM $MgCl_2$ (Alfa Aesar, #J61014) using a 1:10 scaffold/staples ratio and 1:100 for modified staples. Unmodified staples as well as staples internally modified with Cy5 (iCy5, product #1476) were purchased from Integrated DNA Technologies, INC, and biotin-functionalized were purchased from Biomers GmbH. The scaffold and staples mix were initially heated and held at 70 ˚C for 5 min before being cooled to 20 ˚C using a 20 min/1 ˚C linear ramp. A 1% agarose gel electrophoresis (Agarose LE, Biozym Scientific GmbH) was used as a purification procedure to remove the excess of staple strands (1× TAE 12 mM $MgCl_2$ running buffer, 4 V/cm, 1.5 h) while being cooled in an ice water bath. After electrophoresis, the bands in the gel containing the DNA origami structure were cut out and squeezed with a glass slide covered in parafilm to extract the purified DNA origami structures. The final concentration of the DNA origami structures was determined on a Nanodrop 2000 spectrophotometer (Thermo Fisher Scientific). It is important to note that the DNA structures were not stained.

Nanoantenna self-assembly

To functionalize commercially available, citrate-capped 60 nm Au NPs (BBI) with thiolated ssDNA (5'SH -T8 and 5'SH -T18 in 4:1 ratio, Ella Biotech GmbH), disulfide bonds are cleaved with tris (2-carboxyethyl) phosphine at room temperature for 1 h. Then, the cleaved oligonucleotides are mixed with Au NPs and left in a freezer at −20 ˚C for 3 h. After being

thawed, Au NPs are purified from the aggregates and an excess of DNA strands by gel electrophoresis (1× TAE 12 mM $MgCl_2$ running buffer, 4 V/cm, 1.5 h, on ice water bath). The Au NPs are recovered by cutting the band with the highest electromobility. For the DNA functionalization of the super-spherical NPs we followed the same procedure.

Purified origamis and freshly functionalized Au NPs are mixed in a 1:10 molar ratio, and 600 mM NaCl is added. Then, this mixture is left overnight in room temperature to allow assembly. Dimers are purified from the rest by gel electrophoresis. This solution is loaded into 1% agarose gel and separated for 4 h on ice water bath. Dimers are recovered by cutting the respective band and then immobilized on a glass substrate.

Sample Preparation and Surface Immobilization

For immobilization of the structures, glass coverslips were first rinsed with water and then cleaned in a UV cleaning system (PSD Pro System, Novascan Technologies, USA) followed by incubation in 3 M KOH for 5 min. After rinsing with 1× PBS (Alfa Aesar, #J75889), the surface was passivated with BSA biotin (0.5 mg/mL, Sigma-Aldrich Chemie GmbH, #a8549) and neutravidin (0.5 mg/mL, Fisher Scientific AG, #10443985), both sequentially incubated for 30 min and washed with 1× PBS buffer. Then, an additional wash using the origami buffer (1× TAE 12 mM $MgCl_2$) prepared the surface for the incubation. For the control sample, 15 pM of the DNA origami structure was immobilized via biotin binding to the functionalized surface. For the immobilization of the OAs, after incubation with neutravidin the surface was passivated with 500 nM of ssDNA modified with biotin (biotin-A15 Ella Biotech GmbH), for 15 min and then washed with origami buffer (1× TAE 12 mM $MgCl_2$). After 15 min of incubation in case of origami, and 30 min in case of antennas, the sample was washed, and the buffer was

exchanged to a buffer containing coupled glucose oxidase and catalase (GODCAT) in a buffer containing 1 mM glucose in order to increase the photostability of the fluorophores.

Widefield measurements

Measurements were performed on a home-build TIRF wide-field microscope built on an inverted Olympus IX83 body. For excitation, a 640 nm laser (Laserquantum, gem640) is used. Spectral clean-up of the laser's emission is performed through the filters (ZET532/640, Chroma Technology Corporation). Dichroic Mirrors (DM) are used to join the path and πShaper (VIS Flat Top Beam Shaper | πShaper 6_6_VIS) is used providing a flat beam profile suitable for scenarios requiring uniform illumination. Linear polarizer and λ/4 plates (B-Halle, # RAC 3.4.10) were included in the path for circular excitation polarization. The laser light is then focused by two lenses (AC508-100-A-ML and AC254-030-A-ML) into the back focal plane of the UPLAPO100xOHR (1.5 NA, Olympus) objective. A Laser Dual Band Set (ET- 532/640 nm, DM3) is used to reflect the excitation towards the sample and transmit the emission to reach the CMOS camera (C14440 ORCA-Fusion, Hamamatsu). Data acquisition is performed using the open source microscopy imageJ software Micro-Manager[3].

Supplementary Figure 1

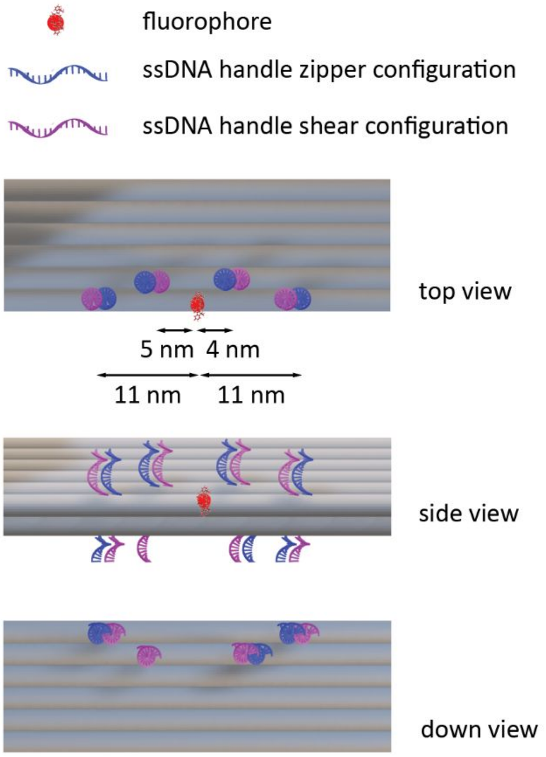

Figure S1. *Top, side and down view of a part of the DNA origami host structure. The positions of the ssDNA "handles" for binding Au NPs, in zipper and in shear configuration are highlighted together with the location of the Cy5 fluorophore employed.*

Supplementary Figure 2

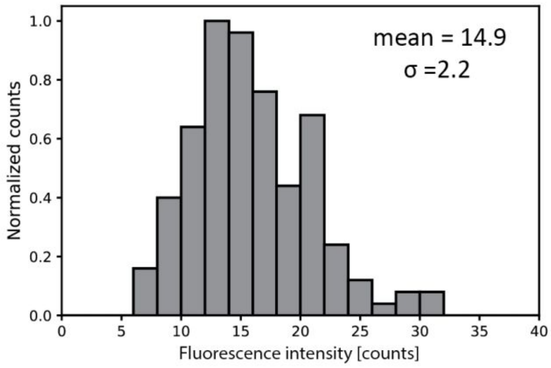

***Figure S2**. Fluorescence intensity histogram plot for the reference sample consisting of a DNA origami structure with a single Cy5 fluorophore and no NPs. 140 structures were measured with the same parameters as the OAs.*

Supplementary Note 2: Log-normal Distributions

The fluorescence enhancement (FE) was determined by normalizing all OAs´ intensity values to the mean intensity of the corresponding reference measurement extracted from Figure S2. To ensure equal consideration of extreme FE values, the distributions for each sample are depicted logarithmically and fitted with a log-normal distribution equation (1):

$$P(x) = \frac{A}{wx\sqrt{2\pi}} e^{\frac{-ln\,(\frac{x}{x_c})^2}{2w^2}} \quad (1)$$

where x_C is the position that maximizes $P(x)$, w represents the ln standard deviation and A is the total area of the $P(x)$ function. The mean value (μ) and the standard deviation (σ) of $P(x)$ are calculated using the following equations (2) and (3) respectively:

$$\mu = x_c\, e^{\frac{w^2}{2}} \quad (2)$$

$$\sigma = \sqrt{e^{w^2}x^2(e^{w^2}-1)} \tag{3}$$

The standard error (SE) is computed using equation (4), where N represents the total number of measured molecules:

$$SE = \frac{\sigma}{\sqrt{N}} \tag{4}$$

Table S1. Parameters from fits (x_c, w, μ, σ, and SE) and measurements (N)

Value \ Sample	Sample Fluo. oriented parallel (‖)	Sample Fluo. oriented perp. (⊥)
x_C	157.6	28.8
w	0.9	1.0
σ	292.7	60.5
μ	245.3	46.9
SE	13.4	3.0
N	479	404

Supplementary Note 3: Super-spherical NPs results

In this supplementary note, we present the results obtained from experiments conducted with "super-spherical" 50nm Au NPs that exhibit significantly lower dispersion in size and shape than standard commercial spherical NPs[4]. These super-spherical NPs were utilized to explore potential differences in FE compared to commercially obtained 60 nm NPs, with the aim of minimizing dispersion in FE results attributable to particle size and uniformity variations. Following the same procedures as with the commercial NPs, all experiments were replicated using the super-spherical NPs. For the samples where the fluorophore was oriented parallel and perpendicular to the antenna's longer axis, 271 and 321 structures were measured, respectively. These structures were later colocalized with SEM images, similar to the process with 60nm NPs. An SEM picture for these structures is presented in Figure S3. Due to their smaller size,

OAs based on these super-spherical nanoparticles are expected to yield lower fluorescence enhancement values as compared to the 60 nm Au NPs.

The FE results are depicted in Figure S4. The average enhancement for the fluorophore oriented parallel and perpendicular to the antenna's longer axis is 35 and 17, respectively. Interestingly, despite employing these well-defined super-spherical NPs, the FE standard deviation remained comparable to the values obtained with the standard commercial NPs suggesting that particle shape and size may not be the critical factors influencing the performance of our OA design – see Supplementary Table 2. This observation underscores the complexity of FE in plasmonic systems and highlights the need for further research to elucidate the underlying mechanisms governing OA functionality.

Supplementary Figure 3

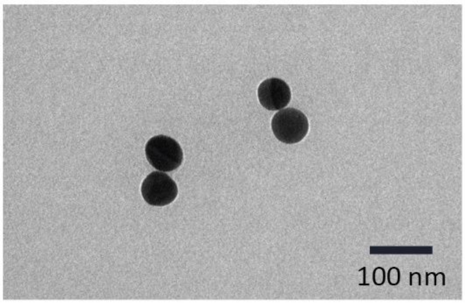

Figure S3. *TEM image of two super-spherical 50 nm Au OAs dimers.*

Supplementary Figure 4

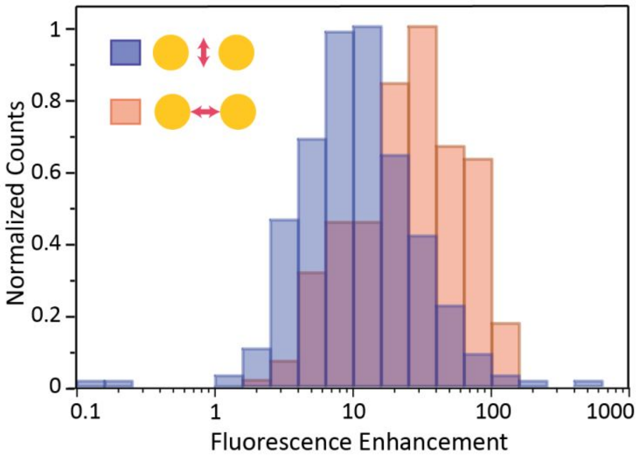

Figure S4. *Fluorescence enhancement histogram plot for super-spherical 50 nm Au OAs dimers with a single Cy5 fluorophore located at the hotspot oriented predominantly parallel (orange) or perpendicular (blue) to the main dimer axis.*

Table S2. Parameters from fits for super-spherical NPs

Value \ Sample	Sample Fluo. oriented parallel (‖)	Sample Fluo. oriented perp. (⊥)
x_C	35.0	9.7
w	1.2	0.8
σ	113.0	12.6
μ	68.0	13.4
SE	6.8	0.7
N	271	321

Supplementary Note 4: Finite element method simulations

The experimentally determined polar angular distributions for the parallel and perpendicular samples (Figure S6) were employed as an input for the numerical simulations[5]. In brief, the

trend curves of quantum yield and excitation enhancement with varying polar angle η are first obtained by simulating for several orientations. The fluorescence enhancements of each fluorophore orientation in the experimental polar angular distributions (Figure S6) can then be determined through the corresponding η angles. Last, these enhancement values were used to obtain the corresponding fluorescence enhancement histograms.

3D fullwave simulations were carried out using the frequency domain solver of the CST Studio Suite software. The "local" mesh setting was used for the Au NPs and the adaptive mesh refinement was turned on in all simulations. The dielectric function of gold was taken from fitting data of Johnson & Christy[6]. The distance between the glass substrate ($\varepsilon = 2.25$, with dimensions of 1000 nm × 1000 nm × 500 nm for length, width, and thickness, respectively) and the surface of the Au NPs was set to 10 nm. To simulate a realistic scenario in which an antenna is deposited onto a glass coverslip, the boundary condition "open (add space)" is applied to the side facing the substrate, while the other boundary settings are left as "open" only. The background permittivity is assumed to be water ($\varepsilon = 1.77$).

To calculate the enhancement factor, a Hertzian dipole (made of PEC material) connected to a discrete port with a resistance of 5000 ohms, was employed to simulate an electric dipole emitter. The dipole was positioned 1.75 nm away from the center of the gap and in proximity to a single Au NP. We assumed that the interaction with the OA doesn't change the intrinsic non-radiated decay rate (γ_{nr0}) in Cy5 (quantum yield $q_0 = 0.3$), and therefore $\gamma_{nr} = \gamma_{nr0}$. This is typically the case within the weak-coupling regime. Then the enhancement of the emitter´s quantum yield is[7]:

$$\frac{q}{q_0} = \frac{\frac{\gamma_r}{\gamma_r+\gamma_{ET}+\gamma_{nr}}}{q_0} = \frac{\frac{\frac{\gamma_r}{\gamma_{r0}}}{\frac{\gamma_r}{\gamma_{r0}}+\frac{\gamma_{ET}}{\gamma_{r0}}+\frac{\gamma_{nr}}{\gamma_{r0}}}}{q_0} = \frac{\frac{\int_0^\infty f_0(\omega)g_r(\omega)d\omega}{\int_0^\infty f_0(\omega)g_r(\omega)d\omega+\int_0^\infty f_0(\omega)g_{ET}(\omega)d\omega+\left(q_0^{-1}-1\right)}}{q_0}, \tag{5}$$

The γ_{ET} and γ_r represent the energy transfer and radiative rates, respectively, of Cy5 in the presence of the OAs, while γ_{r0} is Cy5´s intrinsic radiative rate. In equation (5), $g_r(\omega) = \frac{P_r(\omega)}{P_{r0(\omega)}}$ represents the ratio of enhanced radiated power $P_r(\omega)$ to the radiated power $P_{r0(\omega)}$ without the presence of the OAs. Similarly, $g_{ET}(\omega)$ denotes the ratio of energy loss $P_{loss}(\omega)$, due to the presence of antennas, divided by $P_{r0(\omega)}$. The value $f_0(\omega)$ is obtained from the Cy5 emission spectrum F_0 in buffer as $f_0(\omega) = F_0(\omega)/\int_0^\infty F_0(\omega)d\omega$. In addition,

$$\gamma_r = \gamma_{r0}\int_0^\infty f_0(\omega)g_r(\omega)\, d\omega, \tag{6}$$

$$\gamma_{ET} = \gamma_{r0}\int_0^\infty f_0(\omega)g_{ET}(\omega)\, d\omega. \tag{7}$$

The final enhancement factor equals to $\frac{q}{q_0}\frac{|E\cdot p|^2}{|E_0\cdot p_0|^2}$ which represents the product of the enhancement of both the excitation and the emission (quantum yield), see Figure S5. Considering the orientation of the fluorophore in the DNA origami, E is the electric field vector at the position of the Hertzian dipole in the presence of the OAs under the excitation of a circularly polarized plane wave at normal incidence. The p denotes the dipole moment with the same orientation as the fluorophore in the DNA origami structure. Because the main component of the electric field is along the long axis of dimer, $|E \cdot p|^2$ is simplified as $|E_z|^2|p_0|^2cos^2\eta$, where η is the angle between long axis of the dimer and the dye orientation out of plane. The simulations of the electric field enhancement were carried out at 640 nm to match the laser used

in the experimental setup whereas for the quantum yield simulations, the 620 to 795 nm range was considered to cover for the whole emission range of Cy5.

Supplementary Figure 5

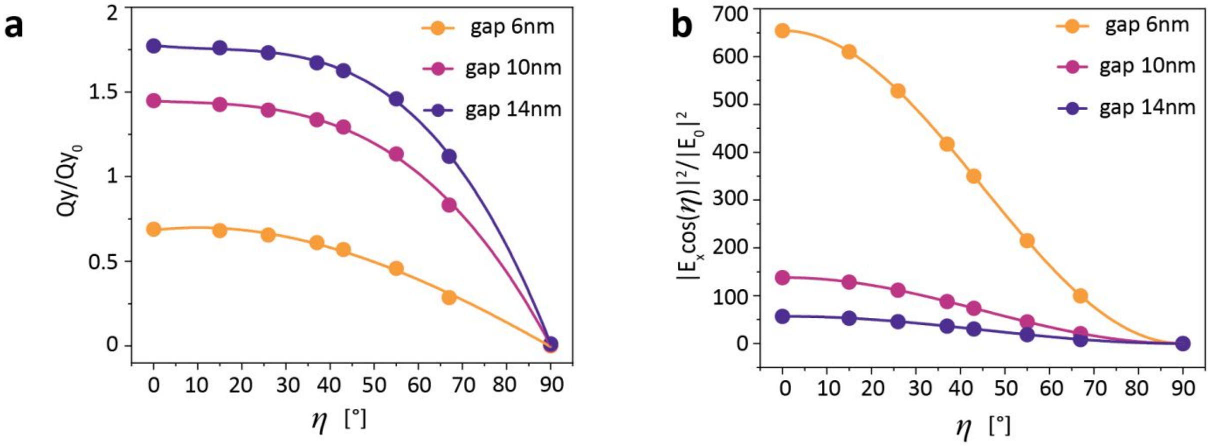

Figure S5. *Simulated fluorescence quantum yield (a) and electric field intensity enhancement as a function of the fluorophore orientation for three different systems with interparticle distances equal to 6 nm, 10 nm, and 14 nm*

Supplementary Figure 6

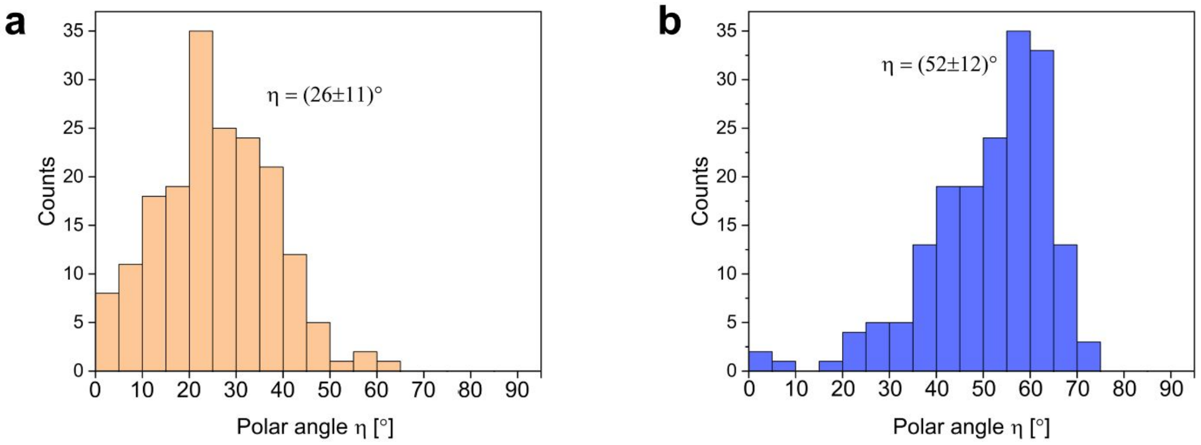

Figure S6. *Experimentally determined polar angle distributions for the parallel (a) and perpendicular samples (b). Their respective median and corresponding standard deviations are (26±11)° and (52±12)°. All the data included in (a) and (b) was previously obtained*[5].

Supplementary Note 5: Consideration of the interparticle gap

The hybridization between DNA strands of poly-T-functionalized 60 nm Au NPs and poly-A handles of DNA origami structures were realized using both zipper and shear configurations[8], as presented in Figure S1. In such a way we were able to increase the number of handles used for the binding around a fluorophore, thus reducing the freedom of movement of Au NPs along the DNA origami surface, as well as increasing the dimer yield. However, the use of two different sequences for the binding can slightly increase the gap variation between individual dimers and make a prediction of the interparticle distance more challenging.

Supplementary Figure 7

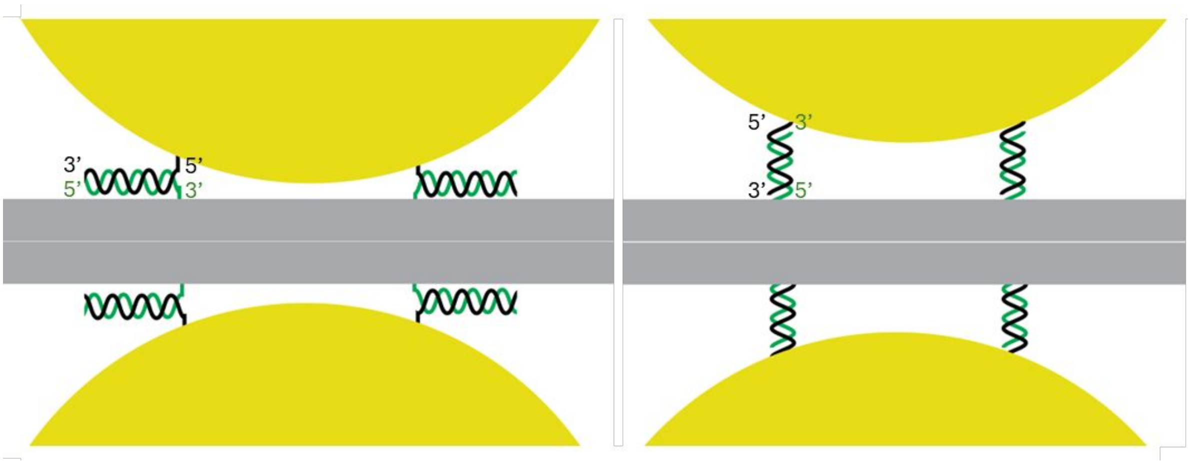

***Figure S7**. Schematic representation of a double helix formed between DNA strands on Au NPs (black) and complementary DNA origami handles (green) with the zipper (left) and the shear configuration (right).*

The difference between the zipper and the shear configuration of hybridization is schematically presented in Figure S7. In the first approach, the poly-A handles are extended out of the DNA origami structures with their 5' end. As Au NPs are covered with the DNA sequences functionalized with a thiol moiety at the 5' position, the hybridization tends to pull the nanoparticles close to the surface of the DNA origami structures. Therefore, we predict a rather small interparticle gap of around 6 nm (5 nm of the DNA origami width + 0.5 nm distance between each nanoparticle and the DNA origami) using zipper handles. The assessment of the dimer gap in case of the shear configuration, when the DNA handles are extended at their 3' end, is however more complex and should be considered in more detail. In general, the DNA origami handles are extended by 15 adenines at a certain distance from the geometrical center of the dimer to realize the binding (see Figure S1 and S8a). Because of the flexibility of the ssDNA, the extension can however freely rotate in respect to the DNA origami surface, thus can bind to the different DNA strands on the nanoparticles at the distance "x" from the DNA origami extension. In such a way, the distance between the Au NPs and the DNA origami surface can vary, depending on the DNA strands of Au NPs that hybridize with the DNA origami handles, as presented in Figure S8b and S8c. Knowing the distance of the handles from the center (a), the length of the hybridizing DNA strands (b), and the NP radius (r), one can calculate the distance of the NP from the DNA origami surface (d) using the following equations:

$$d = y - z \tag{8}$$

$$y = \sqrt{b^2 - x^2} \tag{9}$$

$$z = r^2 - \sqrt{r^2 - (a - x)^2} \tag{10}$$

, which is further explained in Figure S8d.

Using our DNA origami with handles located 11 nm from the center of the dimer and a nanoparticle-DNA origami junction of 6.9 nm (5.1 nm of ds-DNA and 1.8 nm of ssDNA strand), we can expect an intermolecular gap (g) of 6 to 15 nm to be formed in the 60 nm Au dimer, as shown in Figure S8e. Thus, the final gap size is the interplay between the shear configuration that can vary depending on the hybridization position, and the zipper configuration that tend to pull the NPs close to the DNA origami surface. It also means that the thermodynamically preferred interparticle distance of individual dimers is different depending on the way that the hybridization has been realized. Finally, to fully describe the system one must take into consideration steric and electrostatic repulsion between DNA strands on the surface of Au NPs and the ones of the DNA origami surface at short distances[9]. For this reason, to analyze obtained fluorescence intensity distribution, and for the simulations we assumed that the interparticle gap can range from the shortest distance (6 nm) of the only-zipper configuration or the shear configuration with big $|x|$ to the biggest distance (14 nm) realized with no-zipper configuration and the shear configuration with small $|x|$, resulting in the mean gap size of $g = (10 \pm 4)$.

Supplementary Figure 8

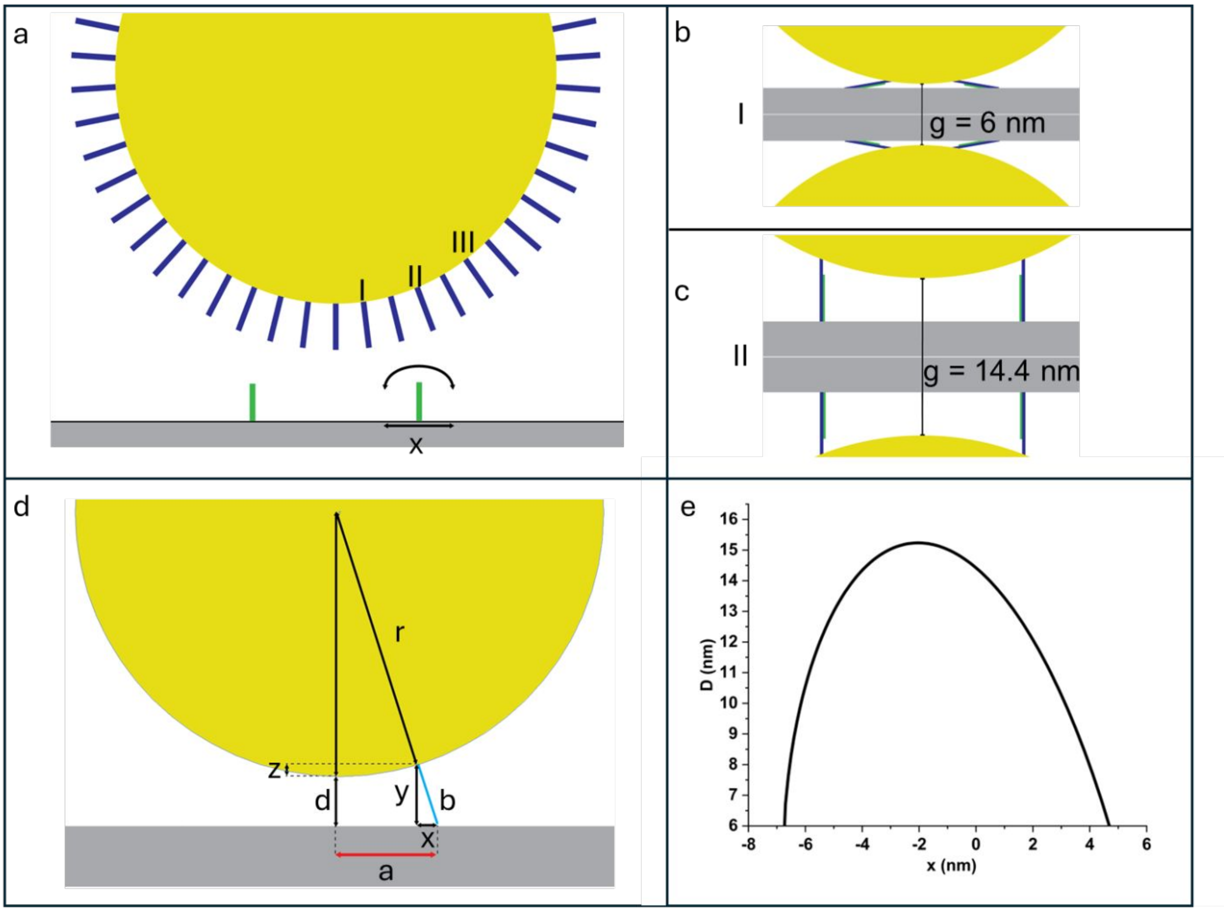

Figure S8. Dependance of the interparticle gap on the hybridization position of the shear configuration. (a) Schematic representation of the binding between DNA origami handles (green) and DNA on Au NPs (blue). The DNA origami handles are ssDNA extensions located at the certain position from the dimer center that can freely rotate in respect to the DNA origami surface (x). In such a way the handles can hybridize with the DNA of Au NPs at different positions (e.g. I, II, or III). (b), (c) A dimer gap after the hybridization of DNA origami handle with the complementary sequence at the position I and II resulting in 6 and 14.4 nm dimer gap respectively. (d) Scheme of the dimer gap calculations. The DNA origami handles are located at the distance "a" from the dimer geometrical center. The ds DNA connecting DNA origami of 5nm and Au NPs at position "x" is represented by a blue line. (e) Dependance of the

interparticle gap (g) on the hybridization position (x) for the handles positioned 11 nm from the dimer center.

References

(1) Douglas, S. M.; Marblestone, A. H.; Teerapittayanon, S.; Vazquez, A.; Church, G. M.; Shih, W. M. Rapid Prototyping of 3D DNA-Origami Shapes with caDNAno. *Nucleic Acids Research* **2009**, *37* (15), 5001–5006. https://doi.org/10.1093/nar/gkp436.

(2) Poppleton, E.; Mallya, A.; Dey, S.; Joseph, J.; Šulc, P. Nanobase.Org: A Repository for DNA and RNA Nanostructures. *Nucleic Acids Research* **2022**, *50* (D1), D246–D252. https://doi.org/10.1093/nar/gkab1000.

(3) Edelstein, A.; Amodaj, N.; Hoover, K.; Vale, R.; Stuurman, N. Computer Control of Microscopes Using µManager. *Current Protocols in Molecular Biology* **2010**, *92* (1), 14.20.1-14.20.17. https://doi.org/10.1002/0471142727.mb1420s92.

(4) Yoon, J. H.; Selbach, F.; Langolf, L.; Schlücker, S. Ideal Dimers of Gold Nanospheres for Precision Plasmonics: Synthesis and Characterization at the Single-Particle Level for Identification of Higher Order Modes. *Small* **2018**, *14* (4), 1702754. https://doi.org/10.1002/smll.201702754.

(5) Adamczyk, A. K.; Huijben, T. A. P. M.; Sison, M.; Di Luca, A.; Chiarelli, G.; Vanni, S.; Brasselet, S.; Mortensen, K. I.; Stefani, F. D.; Pilo-Pais, M.; Acuna, G. P. DNA Self-Assembly of Single Molecules with Deterministic Position and Orientation. *ACS Nano* **2022**, *16* (10), 16924–16931. https://doi.org/10.1021/acsnano.2c06936.

(6) Johnson, P. B.; Christy, R. W. Optical Constants of the Noble Metals. *Phys. Rev. B* **1972**, *6* (12), 4370–4379. https://doi.org/10.1103/PhysRevB.6.4370.

(7) Ringler, M.; Schwemer, A.; Wunderlich, M.; Nichtl, A.; Kürzinger, K.; Klar, T. A.; Feldmann, J. Shaping Emission Spectra of Fluorescent Molecules with Single Plasmonic Nanoresonators. *Phys. Rev. Lett.* **2008**, *100* (20), 203002. https://doi.org/10.1103/PhysRevLett.100.203002.

(8) Vietz, C.; Lalkens, B.; Acuna, G. P.; Tinnefeld, P. Functionalizing Large Nanoparticles for Small Gaps in Dimer Nanoantennas. *New J. Phys.* **2016**, *18* (4), 045012. https://doi.org/10.1088/1367-2630/18/4/045012.

(9) Cui, F.; Marbach, S.; Zheng, J. A.; Holmes-Cerfon, M.; Pine, D. J. Comprehensive View of Microscopic Interactions between DNA-Coated Colloids. *Nat Commun* **2022**, *13* (1), 2304. https://doi.org/10.1038/s41467-022-29853-w.
